# Evaluation of health surveillance system attributes: the case of neglected tropical diseases in Kenya

**DOI:** 10.1186/s12889-021-10443-2

**Published:** 2021-02-23

**Authors:** Arthur K. S. Ng’etich, Kuku Voyi, Clifford M. Mutero

**Affiliations:** 1 School of Health Systems and Public Health (SHSPH), University of Pretoria, Pretoria, South Africa; 2 University of Pretoria Institute for Sustainable Malaria Control (UP ISMC), University of Pretoria, Pretoria, South Africa; 3 International Centre of Insect Physiology and Ecology, Nairobi, Kenya

**Keywords:** Surveillance system, Neglected tropical diseases, Simplicity, Acceptability, Stability, Flexibility, Usefulness, Reporting rates

## Abstract

**Background:**

Control of preventive chemotherapy-targeted neglected tropical diseases (PC-NTDs) relies on strengthened health systems. Efficient health information systems provide an impetus to achieving the sustainable development goal aimed at ending PC-NTD epidemics. However, there is limited assessment of surveillance system functions linked to PC-NTDs and hinged on optimum performance of surveillance system attributes. The study aimed to evaluate surveillance system attributes based on healthcare workers’ perceptions in relation to PC-NTDs endemic in Kenya.

**Methods:**

A cross-sectional health facility survey was used to purposively sample respondents involved in disease surveillance activities. Consenting respondents completed a self-administered questionnaire that assessed their perceptions on surveillance system attributes on a five-point likert scale. Frequency distributions for each point in the likert scale were analysed to determine health workers’ overall perceptions. Data was analysed using descriptive statistics and estimated median values with corresponding interquartile ranges used to summarise reporting rates. Factor analysis identified variables measuring specific latent attributes. Pearson’s chi-square and Fisher’s exact tests examined associations between categorical variables. Thematic analysis was performed for questionnaire open-ended responses.

**Results:**

Most (88%) respondents worked in public health facilities with 71% stationed in second-tier facilities. Regarding PC-NTDs, respondents perceived the surveillance system to be simple (55%), acceptable (50%), stable (41%), flexible (41%), useful (51%) and to provide quality data (25%). Facility locality, facility type, respondents’ education level and years of work experience were associated with perceived opinion on acceptability (*p* = 0.046; *p* = 0.049; *p* = 0.032 and *p* = 0.032) and stability (*p* = 0.030; *p* = 0.022; *p* = 0.015 and *p* = 0.024) respectively. Median monthly reporting timeliness and completeness rates for facilities were 75 (58.3, 83.3) and 83.3 (58.3, 100) respectively. Higher-level facilities met reporting timeliness (*p* < 0.001) and completeness (*p* < 0.001) thresholds compared to lower-level facilities.

**Conclusion:**

Health personnel had lower perceptions on the stability, flexibility and data quality of the surveillance system considering PC-NTDs. Reporting timeliness and completeness rates decreased in 2017 compared to previous surveillance periods. Strengthening all surveillance functions would influence health workers’ perceptions and improve surveillance system overall performance with regard to PC-NTDs.

**Supplementary Information:**

The online version contains supplementary material available at 10.1186/s12889-021-10443-2.

## Background

Health systems comprise of a network of individuals, organised entities and functions with the primary goal of promoting, restoring and sustaining health [1]. Six core components ensure health systems are functional and meet their objectives. They include adequate health service delivery, health workforce development, functional health information systems, equitable access to drugs and technologies, effective health financing and efficient leadership and governance [2]. Particularly, reliable health information systems are pivotal for efficient production and timely utilisation of information on determinants of health, health status and health system performance [3]. These systems provide an evidence-based framework for collection, analysis and interpretation of data to keep policy makers informed on disease occurrence patterns and relevant health determinants [4]. During the 48th World Health Organization (WHO) regional committee in Africa in 1998, the Integrated Disease Surveillance and Response (IDSR) strategy was conceived [5]. To date, tremendous efforts have been achieved since adoption of the strategy in enabling all levels of the health care system to detect, confirm and respond to public health events in order to reduce high levels of mortality, illness and disability in Africa [6]. The IDSR system was a regional strategy to strengthen and improve surveillance systems and use of data for public health actions and response at all levels of the national system [6]. The strategy was adopted by all 47 countries in the WHO African region as from 1998, with Kenya adopting it in 2000 and commencing full implementation in 2006 [6, 7]. The IDSR framework in Kenya classifies neglected tropical diseases (NTDs) into two broad categories as either diseases targeted for elimination, or of public health importance [6].

NTDs are accorded low priority within health strategies as evidenced by the minimal attention they receive from policy makers and consequently, insufficient resource allocation to support research and control [8]. In spite of increased efforts to strengthen health systems, developing nations still struggle to achieve the ultimate goal of NTDs elimination. Well-functioning health systems are a key prerequisite to the achievement of the long-term goal of NTDs elimination and forestalling their re-emergence [9]. Thus, successful push for prevention and control of NTDs spearheaded by the WHO would depend on effective implementation of key health system components [3]. Achieving NTDs elimination targets and halting disease transmission, therefore, requires re-assessment of the effectiveness of existing surveillance, diagnostic and treatment interventions [9, 10].

Efficient health systems provide an impetus towards meeting specific NTD targets in the context of achieving the third sustainable development goal [11]. Transitioning from control to elimination of these diseases necessitates surveillance and response systems to be of priority in health interventions [12]. In this regard, periodical assessments are needed to increase the performance and efficiency of surveillance and response systems and, more importantly, to enhance surveillance data accuracy in describing disease patterns [13]. Naturally, reliable surveillance information would ensure timely public health action and limit the impact of diseases on the affected communities [10].

Previous studies of surveillance and response systems in the African region have focused on assessment of notifiable diseases surveillance systems and the IDSR system, with a leaning on priority diseases [4, 14–17]. Surveillance system assessments concerning NTDs are meagre given low priority and an underestimation of their impact on population health. In Kenya, these diseases remain “neglected” and there are calls for greater recognition of their importance according to the Second National Strategic Plan for Control of Neglected Tropical Diseases. The strategic plan specifically underscores, the need to ensure NTDs are notifiable on the Integrated Disease Surveillance and Response Unit (IDSRU) platform [18].

Assessment of the effectiveness of surveillance and response systems considers surveillance attributes performance among other key surveillance aspects [19]. An effective disease surveillance system improves the ability of a health system to respond to diseases prone to outbreaks [14]. Therefore, frequent assessments of surveillance functions are required to ascertain their performance and users’ perceptions on the surveillance system attributes [14]. However, there are limited systematic assessments of surveillance system attributes based on health workers’ perceptions at the sub-national level in Kenya, which justifies the need for such an assessment with a focus on NTDs. Surveillance system evaluations in developed countries give priority to assessment of surveillance attributes [17, 20, 21], while resource-constrained countries still attempt to address challenges of having functional surveillance core and support functions [22–24]. Certainly, health workers’ perceptions on surveillance system attributes would provide a clear perspective on the overall performance of other surveillance functions. A preceding study conducted in Kenya assessed surveillance core and support functions relating to preventive chemotherapy targeted neglected tropical diseases (PC-NTDs) [25]. Thus, assessment of health care personnel perceptions regarding attributes of the existing surveillance system would be pertinent to obtaining crucial evidence for improving PC-NTDs surveillance and response at the sub-national level. Therefore, this study aimed to evaluate health surveillance system attributes with regard to PC-NTDs considering healthcare workers’ perceptions.

## Methods

### Study setting

The study areas comprised of NTD endemic counties in Kenya, with sub-counties having at least three co-endemic PC-NTDs of known prevalence. The PC-NTDs of focus were Lymphatic Filariasis (LF), Schistosomiasis, Soil Transmitted Helminthiasis (STH) and Trachoma. According to data available in the Second National Strategic Plan for Control of NTDs in Kenya, administrative counties that met this criteria and represented three geographic regions highly endemic for the diseases included: Kwale, Kilifi, Lamu, Tana River and Taita Taveta counties in the Coastal region; Baringo, Narok and West Pokot counties in the Rift Valley region; and, Kitui and Embu counties in the Eastern region [18].

### Surveillance attributes assessment

The study focused on surveillance attribute functions including: (i) simplicity (ease of operation of the surveillance system); (ii) acceptability (willingness of persons to be involved in activities within the surveillance system); (iii) stability (ability of surveillance system to be available and reliable when required); (iv) flexibility (ease of surveillance system to adapt to change of information needs and operating conditions with minimal additional resources); (v) usefulness (surveillance system contribution to control and prevention of adverse health-related conditions); (vi) data quality (accuracy of data collected within the surveillance system), (vii) surveillance data reporting timeliness (how quick information is conveyed across levels of the surveillance system); and, (viii) completeness (expected essential data requirements compared to actual reporting) [26]. Worth noting, is that monthly timeliness reporting rate was expressed as a percentage of total number of reports received on time divided by the total number of reports expected in a month. On the other hand, monthly completeness reporting rate was expressed as a percentage of submitted surveillance reports irrespective of the time the report was submitted divided by the total number of expected monthly reports [6, 27].

### Study design, population and sampling

A descriptive cross-sectional health facility survey was utilised, which involved healthcare personnel responsible for surveillance data collection, collation and transmission. The three study regions comprised of 10 administrative counties prevalent of at least three or more PC-NTDs. Subsequently, 19 sub-counties that were most prevalent of the PC-NTDs were purposively sampled [25]. Health facilities that reported high-threshold levels of PC-NTD cases in the 2017 surveillance period according to data retrieved from the Kenyan Demographic Health Information System (DHIS2) were selected in each of the sub-counties [28]. Hence, 192 health facilities were included in the study and an equivalent number of health facility workers (HFWs) responsible for surveillance data collection and transmission from the selected facilities to the sub-national levels formed the target population.

### Data collection and analysis

A self-administered likert-type questionnaire was used to assess healthcare personnel perceptions on surveillance system attributes relating to the PC-NTDs (Supplementary file 1). The questionnaires gathered respondents’ socio-demographic information and prompted information on respondents’ perceptions of surveillance attributes on simplicity, acceptability, stability, flexibility, usefulness and data quality with regard to PC-NTDs. The questionnaire consisted of at least five questions for each category of surveillance system attributes. The questions were formulated on a five-point likert scale ranging from ‘strongly agree’ to ‘strongly disagree’. In addition, a ‘not applicable’ and ‘don’t know’ options were provided for each question to avoid fence-sitting among responders who would have otherwise opted for an indifferent response [29]. The questionnaire also provided a remarks section for each surveillance attribute to capture further open-ended responses. Data was collected between March 2018 and June 2018. Moreover, surveillance data reporting rates were assessed by retrieving health facility surveillance information on monthly reporting timeliness and completeness from the DHIS2 [28]. Data retrieved included reporting rates for a three-year period (2015, 2016 and 2017), which enabled an assessment of monthly reporting rate trends leading up to the 2017 surveillance period.

Data analysis was undertaken using Stata/IC 14.0 (College Station, 77,845 Texas USA). Frequency distributions for each question in the five-point likert scale were analysed to determine healthcare workers’ overall perceptions concerning PC-NTDs surveillance and response activities. The data on reporting rates were summarised using percentages and estimated median values with the corresponding lower and upper quartiles. Moreover, Pearson’s chi-square and Fisher’s exact tests were used to assess factors associated with each surveillance system attribute in the endemic regions. Thematic analysis was performed for questionnaire open-ended responses regarding all attributes under study. Surveillance system attributes were latent outcome variables derived by analysing a collection of variables in each domain. Internal consistency for the group of variables in each domain was assessed using Cronbach’s Alpha to test for reliability and inter-item correlation. Factor analysis was then implemented for each domain to flag out variables that measured a specific unmeasured attribute. Kaiser-Meyer Olkin (KMO) measure of sampling adequacy was used to assess the factorability of the data. To be factorable, KMO should be at least 0.6 and Bartlett’s test of sphericity should be statistically significant [30]. In this study, KMO was > 0.7 and Bartlett’s measure of sphericity was < 0.001 for all domains thus supporting factorability and sampling adequacy. The number of retained items for each domain was guided by the Kaiser criterion (eigenvalues > 1) and inspection of the scree plot [31]. All the statements with an eigenvalue > 1 and factor loading above 0.4 were retained. The retained variables were further assessed for internal consistency using Cronbach’s Alpha to ascertain that the internal consistency for each domain improved. Furthermore, the retained variables in each domain were summed up and scaled so as to derive a score ranging from 0 to100 using the following formula [32].

$$ \mathbf{Attribute}\ \mathbf{score}=\frac{\mathrm{Actual}\ \mathrm{score}-\mathrm{Minimum}\ \mathrm{score}}{\mathrm{Maximum}\ \mathrm{score}-\mathrm{minimum}\ \mathrm{score}}\times 100\% $$

## Results

### Socio-demographic characteristics

Data was collected from three regions comprising 10 counties: Coast (Kwale, Kilifi, Lamu, Tana River and Taita Taveta), Rift Valley (Baringo, West Pokot and Narok) and Eastern (Kitui and Embu) with county distribution of the respondents ranging from 5 to 18%. The study enrolled 192 respondents, 88% of them worked in public health facilities with a majority (71%) stationed in second tier (dispensary) facilities (Table 1). Data showed that 61% of respondents were aged between 18 and 40 years, 51% were female and 83% had attained at least a diploma level of education. The health cadre for 65, 22 and 10% of respondents were nursing, clinical and public health staff respectively. Two-thirds (126/192) of respondents had worked in their current health cadre for more than 3 years.

**Table 1:** Socio-demographic characteristics of respondents

Characteristic	N	n (%)
Regions		
Coastal (Kwale, Kilifi, Lamu, Tana River and Taita Taveta)		104 (54%)
Rift Valley (Baringo, West Pokot and Narok)	192	42 (22%)
Eastern (Kitui and Embu)		46 (24%)
Health facility type		
Public	192	169 (88%)
Private		23 (12%)
Health facility level		
Level 2 – Dispensary		136 (71%)
Level 3 – Health Centre	192	42 (22%)
Level 4 – Sub County Hospital		12 (6%)
Level 5 – County Referral Hospital		2 (1%)
Age category		
18–30 years		26 (14%)
31–40 years	192	91 (47%)
41–50 years		60 (31%)
> 50 years		15 (8%)
Gender		
Female		98 (51%)
Male	192	94 (49%)
Highest level of education		
Certificate		13 (7%)
Diploma	192	159 (83%)
Degree		20 (10%)
Health cadre		
Nursing officer		125 (65%)
Clinical officer		43 (22%)
Public health officer	192	20 (10%)
Health records and management officer		3 (2%)
Laboratory technologist		1 (1%)
Years of work experience in health cadre		
1- < 2 years		13 (7%)
2- < 3 years	192	53 (28%)
3- ≤ 5 years		56 (29%)
More than 5 years		70 (36%)

*N* Number of respondents with valid data analysed

### Surveillance system attributes

**Fig. 1:**
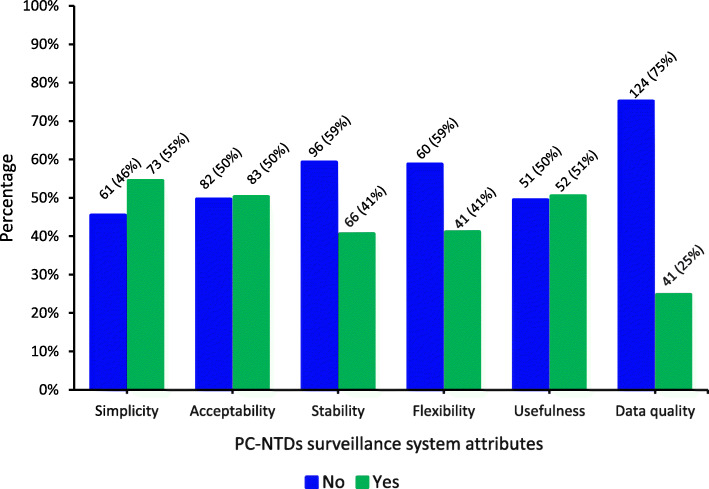
Distribution of respondents’ perceptions on PC-NTDs surveillance attributes

The total number of respondents assessed for each attribute included those that answered in the affirmative (Strongly Agreed/Agreed) or vice-versa (Strongly Disagreed/Disagreed) and those who were neutral (Neither Agreed nor Disagreed) on the likert questions. On the other hand, data from respondents that selected the “Don’t Know” and “Not Applicable” options for each of the likert questions was treated as missing data during analysis and were assumed to be a random sample whose opinion would not change the study outcome. Among respondents that gave valid responses to the likert questions, a binary outcome variable was derived by categorising the scores into two; ≤ 50 and > 50. The cut off score was 50% implying that those respondents with scores above 50 perceived the surveillance system attribute to be sufficiently met while those with scores below or equal to 50 perceived otherwise. Therefore, among respondents with an affirmative opinion on each attribute being met, 55% (73/134) perceived the surveillance system to be simple, 50% (83/165) to be acceptable, 41% (66/162) to be stable, 41% (42/102) to be flexible, 51% (52/103) to be useful and 25% (41/165) indicated that the surveillance system provided quality PC-NTDs surveillance data (Fig. 1).

Data reduction was performed using factor analysis to establish the important indicators for each of the 6 PC-NTDs surveillance system attributes (simplicity, acceptability, stability, flexibility, usefulness and data quality). Prior to and after data reduction, internal consistency of the variables in each domain were assessed (Table 2).

**Table 2:** Surveillance system attributes regarding PC-NTDs

Surveillance attributes	Cronbach’s Alpha prior to actor Analysis	Initial number of variables	Cronbach’s Alpha after Factor Analysis	Final number of variables retained	Number of variables dropped
Simplicity	0.88	15	0.90	12	3
Acceptability	0.79	7	0.79	7	0
Stability	0.85	6	0.85	6	0
Flexibility	0.92	6	0.92	6	0
Usefulness	0.95	11	0.95	11	0
Data quality	0.77	5	0.82	4	1

Factor analysis showed that there was only one unmeasured attribute that the groups of variables in each domain were measuring. Three variables from the group measuring simplicity and one from the group of variables measuring data quality were dropped upon subjecting the variables to factor analysis with the rest of the domains remaining unaffected. Factor analysis demonstrated that the dropped variables were redundant and hardly introduced any variability on the determination of the unmeasured outcomes. Upon dropping the variables, the model yielded higher Cronbach’s Alpha of 0.90 and 0.82 from 0.88 and 0.77 for simplicity and data quality respectively. The attributes that were not reduced demonstrated good internal consistency and dropping any of the individual items would not yield any improvement in the Cronbach’s Alpha score of measuring internal consistency.

### Simplicity

Slightly more than half (58%) and up to 54% of respondents either agreed or strongly agreed that reporting PC-NTDs within the IDSR system and completing reporting forms to capture PC-NTDs surveillance data were simple respectively (Supplementary Figure 1). Respondents with a contrary opinion on the ease of completing reporting forms remarked;

*“Reporting some of the NTDs using the available monthly summary forms is cumbersome at times since these diseases are not listed in the forms and have to be reported as ‘other’ conditions” –* HFW#010 (Baringo County)

*“Health personnel of all cadres require training on completing surveillance reporting forms…this will ensure every health worker is conversant with using the tools when required”* – HFW#022 (West Pokot County)

There was an affirmative opinion from 55% of respondents who either agreed or strongly agreed that the instructions and guidelines for completing surveillance forms for reporting PC-NTDs were easy to understand. Close to half (49%) of the respondents either agreed or strongly agreed that it was easy to understand the functionality of the IDSR system. A further one-fifth (21%) of respondents either agreed or strongly agreed that other health personnel were conversant with the IDSR system. Up to 36% of respondents agreed that the case definitions for PC-NTDs were easily applicable with slightly more than half (57%) of them either disagreeing or strongly disagreeing that the existing IDSR system easily accommodates all PC-NTDs. Nearly a half (49, 43, and 50%) of respondents either disagreed or strongly disagreed that the methods for PC-NTDs surveillance data collection were simple, that PC-NTDs surveillance data were easily managed and that the methods used for the analysis of PC-NTDs surveillance data were simple respectively. Up to 47 and 40% of respondents either disagreed or strongly disagreed that the time spent collecting and analysing PC-NTDs surveillance data was minimal respectively. A further 63% of respondents either disagreed or strongly disagreed that minimal training was required to manage PC-NTDs surveillance data. The follow up process for PC-NTDs surveillance data was not simple according to 56% of respondents who either disagreed or strongly disagreed. Close to half (49%) of the health workers either agreed or strongly agreed that the reporting levels for PC-NTDs surveillance data were minimal. There was evidence from data that perceived simplicity of the surveillance system with regard to PC-NTDs varied significantly across the study regions. Respondents sampled from the Rift Valley region had a lower perception on simplicity of the system compared to respondents in the Eastern and Coastal regions (Rift Valley, 33% vs. Eastern, 61% vs. Coastal, 61%, *p* = 0.022).

### Acceptability

Findings showed that up to 68% of respondents were in agreement (agreed or strongly agreed) that their contributions and inputs to the existing IDSR system were considered valuable (Supplementary Figure 2). The opinion of 47% of respondents demonstrated satisfaction with their involvement in PC-NTDs surveillance activities at the facility level. Half (51%) of the respondents either agreed or strongly agreed that fellow health facility workers demonstrated interests in PC-NTDs surveillance activities and 39% were in agreement that the health facility management adequately supported the surveillance activities. Up to 43% of health facility workers agreed PC-NTDs were considered of public health importance in the region with further results showing that two-thirds (66%) of respondents either agreed or strongly agreed that the community in the region supported PC-NTDs surveillance activities undertaken by the facility.

*“Only trachoma is considered of public health importance in the region and there lacks a linkage between the facility and community…so they* (community) *hardly support or get involved in surveillance activities”* – HFW#025 (West Pokot County)

*“More health education and sensitisation at the community level on surveillance activities for NTDs is required to encourage community participation in surveillance activities”* – HFW#041 (Narok County)

Up to 80% of respondents either agreed or strongly agreed that the existing IDSR system protected the privacy and confidentiality of the users. Respondents in the Rift Valley region had lower perceptions on acceptability of the surveillance systems concerning PC-NTDs compared to respondents in the Eastern and Coastal regions (Rift Valley, 32% vs. Eastern, 54% vs. Coastal, 56%, *p* = 0.046). Further, respondents sampled from private facilities were more likely to perceive the surveillance system as acceptable compared to their counterparts in public facilities (74% vs. 47%, *p* = 0.049). Health care workers with advanced level of training were less likely to perceive the surveillance system as acceptable (Certificate, 90% vs. Diploma, 49% vs. Degree, 42%, *p* = 0.032) and those with advanced years of work experience were more likely to perceive the surveillance system as acceptable (1- < 2 years, 60% vs. 2- < 3 years, 38% vs. 3- ≤ 5 years, 43% vs. more than 5 years, 64%, *p* = 0.032).

### Stability

One third (32%) of respondents either agreed or strongly agreed that the existing IDSR system had always been reliable when reporting PC-NTDs surveillance data while 34% of health workers concurred that surveillance forms for reporting PC-NTDs were always available when required (Supplementary Figure 3).

*“Reporting systems for NTDs need to be established and strengthened by providing adequate reporting forms and frequently training health workers on the surveillance activities”* – HFW#063 (Kwale County)

A further one third (33%) of respondents either agreed or strongly agreed that difficulties experienced within the IDSR system concerning PC-NTDs were addressed with minimal delays. Up to 43% of respondents either agreed or strongly agreed that PC-NTDs surveillance activities were adequately supported by those overseeing disease surveillance and response activities in the region. Slightly more than two-thirds (68%) of respondents either disagreed or strongly disagreed that the resources provided to facilitate PC-NTDs surveillance and response activities at the health facility or in the region were sufficient.

*“The transport costs we incur when submitting monthly surveillance data in person is a major challenge…there is not enough financial support for reporting surveillance data”* – HFW#011 (Baringo County)

Further, two-thirds (67%) of respondents either agreed or strongly agreed that the PC-NTDs surveillance data and records storage in the facility was safe and efficient. Respondents in the Rift Valley region had a lower perceived opinion on the stability of the surveillance systems in relation to PC-NTDs compared to respondents in the Eastern and Coastal regions (Rift Valley, 22% vs. Eastern, 43% vs. Coastal, 48%, *p* = 0.030). Further, health workers in private facilities were more likely to perceive the surveillance system as being stable compared to those in public facilities (67% vs. 38%, *p* = 0.022). Respondents with advanced level of training were less likely to perceive the surveillance system as being stable (Certificate, 88% vs. Diploma, 37% vs. Degree, 47%, *p* = 0.015) and those with more years of work experience were more likely to perceive the surveillance system as stable in view of PC-NTDs surveillance activities (1- < 2 years, 30% vs. 2- < 3 years, 28% vs. 3- ≤ 5 years, 38% vs. more than 5 years, 56%, *p* = 0.024).

### Flexibility

Health workers (27%) either agreed or strongly agreed that the existing surveillance reporting system was well adapted to reporting all the endemic PC-NTDs in the region (Supplementary Figure 4). Slightly more than a quarter (27%) of respondents either agreed or strongly agreed that PC-NTDs surveillance and response was efficiently achieved within the IDSR system. Up to 43% of respondents either disagreed or strongly disagreed that the existing IDSR system easily adapted to changes in PC-NTDs information needs such as changes in standard case definitions. A further 35% of respondents failed to agree that PC-NTDs surveillance activities within the existing IDSR system easily adapted to changes in funding in the region. Up to 31% of healthcare workers either agreed or strongly agreed that PC-NTDs surveillance activities easily adapted to changes in technology.

*“Possibilities of adapting new reporting technologies along paper-based reporting forms would make reporting of NTDs easier and timely”* – HFW#060 (Kwale County)

The proportion of respondents who either agreed or strongly agreed that the existing IDSR system could easily adapt to new PC-NTDs surveillance data sources was 29%. Data showed no evidence of association between respondents’ perceived opinion on flexibility of the surveillance system in relation to PC-NTDs and their socio-demographic characteristics (*p* > 0.05). However, health workers in private facilities were more likely to perceive the surveillance system as flexible considering PC-NTDs surveillance activities compared to their counterparts in public health facilities (77% vs. 36%, *p* = 0.007).

### Usefulness

Respondents (38%) either agreed or strongly agreed that PC-NTDs surveillance activities had enabled achievement of surveillance objectives in the previous year (Supplementary Figure 5). A further 41% either agreed or strongly agreed that PC-NTDs surveillance data had informed program implementation for disease control in the previous year. Less than one-third (31%) of respondents agreed or strongly agreed that PC-NTDs surveillance data had stimulated research-related activities in the region.

*“We are not aware if surveillance data generated from our facilities stimulates research or attracts funding from donors…so far we have not received any feedback concerning such activities”* – HFW#099 (Kilifi County)

One-quarter (24%) of respondents either agreed or strongly agreed that PC-NTDs surveillance data generated at facility level in the region had attracted donor funding for disease control;

*“Surveillance data generated by facilities in the past gave an estimate of the magnitude of trachoma cases in the region…this attracted funding support from various donors who support projects aimed to control the eye infection”* – HFW#029 (West Pokot County)

Up to 46% of respondents either agreed or strongly agreed that PC-NTDs surveillance and response activities were considered important within the IDSR system. Further, health workers (46%) either disagreed or strongly disagreed that the IDSR system provided sufficient information for prompt public health action to PC-NTDs.

*“I strongly concur that the surveillance reports generated in the past year enabled us take quick actions to respond to probable schistosomiasis outbreaks in the region”* – HFW#072 (Kwale County)

Respondents (38%) further agreed or strongly agreed that surveillance data provided an estimate of PC-NTDs morbidity magnitude in the region. The proportion of respondents who either agreed or strongly agreed that PC-NTDs surveillance data identifies risk factors associated with the diseases was 57%. Moreover, the proportion of respondents that agreed or strongly agreed that PC-NTDs surveillance data detects trends in changes of disease occurrence in the region was 42%.

*“Collection of surveillance data for NTDs common in this area has been helpful…we were able to monitor diseases trends and identify areas with high disease transmission”* – HFW#061 (Kwale County)

Up to 47% of facility workers agreed or strongly agreed that surveillance data collected in the past year enabled PC-NTDs prevention and control programmes impact assessment in the region. Further, there was no evidence of association between respondents’ perceived opinion on usefulness of the surveillance system considering PC-NTDs and their socio-demographic characteristics (*p* > 0.05). However, respondents sampled from private facilities were more likely to perceive the surveillance system as useful in supporting PC-NTDs surveillance activities compared to health workers in public facilities (86% vs. 45%, *p* = 0.008).

### Data quality

Slightly more than a quarter (27%) of respondents either agreed or strongly agreed that missing data was a common occurrence in the forms for reporting PC-NTDs surveillance data (Supplementary Figure 6).

*“Adoption of electronic reporting systems starting from the peripheral levels will ensure quality of surveillance data and reduce instances of missing data”* – HFW#083 (Kilifi County)

Close to half (49%) of the respondents either agreed or strongly agreed that the surveillance forms for reporting PC-NTDs were clear and elaborate. Furthermore, 64% of respondents disagreed or strongly disagreed that the training offered on completion of surveillance reporting forms was adequate.

*“We require regular refresher courses on disease surveillance to generate accurate surveillance data on NTDs common in this area”* – HFW#051 (Kwale County)

*“Data quality on surveillance information can only be achieved if all health workers will be sensitised on proper reporting of the neglected tropical conditions using the available tools and based on the standard case definitions provided in the guidelines”* – HFW#143 (Embu County)

Further, 63% of respondents disagreed or strongly disagreed that supervisory support offered during completion of surveillance reporting forms was adequate. Slightly more than half (52%) of the health workers disagreed or strongly disagreed that the time allocated for PC-NTDs surveillance data management was adequate. Findings showed no evidence of association between respondents’ perceived opinion on data quality of the surveillance system considering PC-NTDs and their socio-demographic characteristics (*p* > 0.05). Nonetheless, respondents in private facilities were more likely to perceive the surveillance system as providing quality PC-NTDs surveillance data compared to health workers in public facilities (58% vs. 21%, *p* = 0.001).

### Timeliness and completeness

Of the 192 health facilities surveyed in PC-NTD endemic regions for the 2017 surveillance period, the median monthly reporting timeliness rates for surveillance reports for persons aged over 5 years and among those aged below 5 years were equivalent; 75 (IQR: 58.3, 83.3). Findings also showed that 38% (73/192) and 36% (69/192) of the facilities met or exceeded the 80% timeliness reporting threshold in both under-fives and above five monthly surveillance reports respectively (Table 3). Similarly, in the same 2017 surveillance period, the median monthly reporting completeness rates for surveillance reports for persons aged over 5 years and among those aged below 5 years were equal; 83.3 (IQR: 58.3, 100). Results further indicated that 56% (107/192) and 54% (104/192) of the facilities met or exceeded the 80% completeness reporting threshold in both under-fives and above five monthly surveillance reports respectively in the same surveillance period. Findings based on submitted monthly surveillance reports for individuals aged over 5 years in the 2017 surveillance period showed that a significantly larger proportion of higher-level facilities (level 3, 4 and 5) met the 80% timeliness reporting threshold compared to lower level 2 facilities (59% vs. 27%, *p* < 0.001). Health facilities with functional laboratories significantly met the 80% timeliness reporting threshold (Yes, 46% vs. No, 25%, *p* = 0.002). Further, availability of electricity (Yes, 39% vs. No, 18%, *p* = 0.031) and availability of computers (Yes, 54% vs. No, 24%, p < 0.001) were associated with meeting the 80% timeliness reporting threshold. On the other hand, submitted monthly surveillance reports for those aged below 5 years in the 2017 surveillance period indicated that a significantly larger proportion of higher-level facilities (level 3, 4 and 5) met the 80% timeliness reporting threshold compared to lower level 2 facilities (57% vs. 30%, *p* < 0.001). Similarly, availability of a functional laboratory (Yes, 48% vs. No, 27%, *p* = 0.003) was associated with meeting the 80% timeliness reporting threshold. Further, availability of electricity (Yes, 42% vs. No, 18%, *p* = 0.017) and computers (Yes, 54% vs. No, 27%, *p* < 0.001) were associated with meeting the 80% timeliness reporting threshold.

**Table 3:** Timeliness and completeness of monthly surveillance data (2017 surveillance period)

Characteristic	N	Under 5 years			Over 5 years		
		Median (IQR)	≥80 (N, %)	< 80 (N, %)	Median (IQR)	≥80 (N, %)	< 80 (N, %)
**Timeliness**	192	75 (58.3–83.3)	73 (38%)	119 (62%)	75 (58.3–83.3)	69 (36%)	123 (64%)
**Completeness**	192	83.3 (58.3–100)	107 (56%)	85 (44%)	83.3 (58.3–100)	104 (54%)	88 (46%)

*N* Number of observations with valid data analysed

*Source data: Kenya Health Information System (KHIS) for Aggregate Reporting* [28]

Regarding reporting completeness rates, submitted monthly surveillance reports for individuals aged over 5 years in the 2017 surveillance period showed that a significantly larger proportion of higher-level facilities (level 3, 4 and 5) met the 80% completeness reporting threshold compared to lower level 2 facilities (80% vs. 43%, *p* < 0.001). Further results showed that the availability of reporting forms (Yes, 57% vs. No, 32%, *p* = 0.025) and presence of a functional laboratory (Yes, 67% vs. No, 40%, *p* < 0.001) were associated with meeting the 80% completeness reporting threshold. Availability of computers (Yes, 70% vs. No, 44%, *p* < 0.001) and availability of surveillance posters (Yes, 60% vs. 45%, *p* = 0.040) were associated with meeting the 80% completeness reporting threshold. Likewise, submitted monthly surveillance reports for those aged below 5 years in the 2017 surveillance period indicated that a significantly larger proportion of higher-level facilities (level 3, 4 and 5) met the 80% completeness reporting threshold compared to lower level 2 facilities (75% vs. 48%, *p* = 0.001).

Comparing monthly reporting rates between the two preceding years to the 2017 surveillance period, findings indicated that there was an 18% (91.7 to 75) decrease in the median reporting timeliness rates in both the under-fives and above five monthly surveillance reports between the 2015 and 2017 surveillance periods (Table 4). Further, reporting timeliness rates in under-fives and above five monthly surveillance reports decreased by 18% (91.7 to 75) and 10% (83.3 to 75) respectively between 2016 and 2017 surveillance periods. On the other hand, there was an equivalent 17% (100 to 83.3) decrease in median reporting completeness rates for the under-fives and above five monthly surveillance reports in both the 2015–2017 and 2016–2017 surveillance periods respectively (Table 5). Further findings indicated that there was a 50% (145 to 73) and 43% (128 to 73) decrease in the number of health facilities that met the 80% timeliness reporting threshold for monthly surveillance reports submitted for those aged below 5 years in the 2015–2017 and 2016–2017 surveillance periods respectively. Additionally, there was a 50% (138 to 69) and 30% (98 to 69) decrease in the number of facilities that met the 80% timeliness reporting threshold for surveillance reports submitted for those aged above 5 years in the 2015–2017 and 2016–2017 surveillance periods respectively (Table 4). On the other hand, there was a 34% (162 to 107) and 37% (171 to 107) decrease in the number of health facilities that met the 80% completeness reporting threshold for surveillance reports submitted for those aged below 5 years in the 2015–2017 and 2016–2017 surveillance periods respectively. Further, there was a 34% (158 to 104) and 33% (156 to 104) decrease in the number of facilities that met the 80% completeness reporting threshold for surveillance reports submitted for those aged above 5 years in the 2015–2017 and 2016–2017 surveillance periods respectively (Table 5).

**Table 4:** Monthly timeliness rates for health facilities in PC-NTDs endemic regions for a three-year period

Year	Under 5 years			Over 5 years		
	Median (IQR)	≥80 (N, %)	< 80 (N, %)	Median (IQR)	≥80 (N, %)	< 80 (N, %)
**2015**	91.7 (83.3–100)	145 (76%)	47 (24%)	91.7 (75–91.7)	138 (72%)	54 (28%)
**2016**	91.7 (75–100)	128 (67%)	64 (33%)	83.3 (75–91.7)	98 (51%)	94 (49%)
**2017**	75 (58.3–83.3)	73 (38%)	119 (62%)	75 (58.3–83.3)	69 (36%)	123 (64%)

*N* Number of observations with valid data analysed

Source data: Kenya Health Information System (KHIS) for Aggregate Reporting [28]

**Table 5:** Monthly completeness rates for health facilities in PC-NTDs endemic regions for a three-year period

	Under 5 years			Over 5 years		
	Median (IQR)	≥80 (N, %)	< 80 (N, %)	Median (IQR)	≥80 (N, %)	< 80 (N, %)
**2015**	100 (91.7–100)	162 (84%)	30 (16%)	100 (91.7–100)	158 (82%)	34 (18%)
**2016**	100 (91.7–100)	171 (89%)	21 (11%)	100 (83.3–100)	156 (81%)	36 (19%)
**2017**	83.3 (58.3–100)	107 (56%)	85 (44%)	83.3 (58.3–100)	104 (54%)	88 (46%)

*N* Number of observations with valid data analysed

Source data: Kenya Health Information System (KHIS) for Aggregate Reporting [28]

## Discussion

The present study findings identified challenges facing the ease of completing IDSR forms when reporting PC-NTDs, understanding surveillance guidelines for completing the forms and utilisation of PC-NTDs standard case definitions. Similarly elsewhere, surveillance manuals were outdated and not easily understood by most health workers [23]. Further comparable findings indicated that IDSR reporting forms completion process was deemed cumbersome and complicated [33]. However, health workers in Northern Ghana admitted that the electronic reporting system had made reporting easier [34]. Contrarily, in a study in South Africa, close to three-quarters of healthcare providers admitted that the processes of the existing surveillance system were simple and comprehensible [14]. Therefore, there is need to provide simplified and understandable standard case definitions and surveillance terms of reference guidelines [35]. Enhanced health worker involvement in routine reporting is dependent on the perceived simplicity of the system. Hence, simplifying training materials will ease understanding and motivate health worker participation in surveillance activities [36]. Our findings showed that respondents had difficulties understanding functionalities of the existing surveillance system with a majority further reporting that their fellow colleagues lacked adequate knowledge on the IDSR system. Therefore, enhanced training of health workers on surveillance system processes ensures ease of understanding of the functionalities [14].

About two-thirds of respondents in the current study perceived their inputs and contributions to activities within the existing surveillance system as being valued. However, less than a half of the health workers were satisfied with their involvement in facility surveillance activities relating to PC-NTDs with the same proportion reporting that their fellow colleagues lacked interest in PC-NTDs surveillance activities. In contrast, elsewhere, majority of health workers were willing to participate in surveillance activities and felt responsible to notify disease conditions [36]. Only about a quarter of the health providers in a study in South Africa were willing to be involved in activities within the existing notifiable disease surveillance system [14]. Further findings indicated that majority of health workers perceived PC-NTDs to be of low priority compared to other conditions, hence they were only willing to engage in surveillance activities of the diseases considered of priority. However, respondents reported that the community levels were more willing to support and be involved in PC-NTDs surveillance activities. Comparably, lack of close involvement in operational activities by health personnel in higher surveillance levels results in overestimation of surveillance system acceptability [14]. Our study further showed a significant association between the number of years worked by respondents in their current cadre and acceptability of the surveillance system. However, findings elsewhere showed that health providers with more working experience were less likely to consider the surveillance system as being acceptable in terms of their willingness to participate in surveillance activities [14].

Health facility workers reported that the existing IDSR system was less flexible in terms of being well adapted to report all co-endemic PC-NTDs, adapting easily to changes in PC-NTDs information needs, technology and funding and efficiently achieving PC-NTDs surveillance within the system amid surveillance activities for other conditions. Comparably, elsewhere, study participants differed on the willingness of health providers to participate in surveillance activities that were dependent on the ability of the existing surveillance system to accommodate changing needs [14]. Current study findings identified minimal adaptability of the existing surveillance system to changes in reporting mechanisms from paper-based to electronic systems at the facility level. Furthermore, health workers expressed a predicament regarding the difficulty of sustaining functional surveillance activities in case of insufficient funding.

There are notable disparities in studies assessing surveillance system stability. System stability is subjective and dependent on the performance of specific measured variables [36]. Our findings depicted that the existing IDSR system in Kenya lacked stability concerning PC-NTDs surveillance data reporting, adequacy of the available forms to report PC-NTDs, resource sufficiency and capacity of health facility managers to support surveillance activities and tackle the challenges encountered with minimal delays. Comparably elsewhere, inadequately trained staff, case definitions unavailability and limited resource capacity proved that the surveillance system was unstable [36]. Insufficient resource capacity renders the system unstable and affects optimal functioning of other surveillance core functions, for instance internet outages and connectivity challenges limit effective submission of surveillance reports through electronic reporting systems [37]. Furthermore, similar to our study findings, inconsistencies in electricity supply hinders adequate and timely submission of surveillance data across surveillance levels [37].

Our findings indicated that less than one-half of respondents perceived PC-NTDs surveillance activities within the IDSR system as lacking usefulness on several fronts in the previous one-year surveillance period. Health workers differed on PC-NTDs surveillance data being sufficient to inform control programs implementation and attracting adequate donor funding. Contrarily, a review of collected malaria surveillance data propelled requests for essential drugs and implementation of control interventions [38]. Likewise, health facility workers in Zimbabwe reported that the existing notifiable disease surveillance system was useful and had previously informed control actions [36]. This signifies lack of prioritisation for neglected conditions within surveillance systems relative to other notifiable conditions. Further findings from our study identified low priority accorded to PC-NTDs within the IDSR system and contrast among respondents on the capacity of the surveillance system to provide sufficient information to inform efficient public health actions to tackle PC-NTDs either at the facility level or regionally. In contrast, about two-thirds of health providers claimed that the notifiable disease surveillance system provided useful information to inform disease control and prevention actions in South Africa [14]. Findings also showed that PC-NTDs surveillance data was not sufficient to provide information on morbidity burdens in the regions, variations in disease occurrence trends and aid control programs impact assessments. Therefore, demonstrating the dearth of PC-NTDs surveillance data to adequately inform programmatic decisions. Elsewhere, surveillance data was useful for monitoring disease activities at the district level [38]. Furthermore, a review of collected surveillance data informed regional level action of reimbursing health workers responsible for submitting IDSR reports to motivate their continued efforts [38]. However, other findings indicated that there was limited utilisation of surveillance data to inform decisions and actions especially at the lower surveillance levels [22]. Notably from our study, more than half of the respondents admitted to the usefulness of surveillance data in identifying PC-NTDs risk factors in the regions. Increased reports of PC-NTD cases informed further investigations on the existing risks factors in the given region. However, major challenges still face utilisation of analysed surveillance data by sub-national level decision makers with regional surveillance levels focusing on meeting reporting deadlines and hardly utilising analysed surveillance data for planning and decision-making [34, 39].

In the present study, direct observations to review accuracy of health facility records for PC-NTDs data was limited due to lack of adequate provision for PC-NTDs in the monthly summary reporting forms. However, most respondents indicated that missing PC-NTDs data in the reporting forms was not common. Elsewhere, missing information in the reporting forms was a common occurrence that affected surveillance data quality [36]. In Ghana, data available in the health facility registers accurately corresponded to the health facility reports submitted to the regional level [40]. Contrarily, in the current study, similar comparisons were not feasible to ascertain data accuracy due to frequent instances of data inconsistencies in health facility registers and retrieval difficulties of hardcopy reports submitted to the sub-national level. Challenges of retrieving actual physical reports across the surveillance levels was due to several reasons including complete failure to send the hardcopy reports, failure to deliver hardcopy reports to the right person or loss of delivered surveillance reports. Some of these reasons were commonly reported in Tanzania and relatable to our study findings [38]. Inefficient data management in terms of poor health records filing and retrieval difficulties posed a major challenge to assessment of PC-NTDs surveillance data reporting rates at the health facility and sub-county levels. Therefore, assessment of reporting timeliness and completeness was entirely based on data retrieved from DHIS2 [28]. This limited our capacity to compare physical records available at specific surveillance levels to data retrieved from DHIS2. Similar findings attributing to weak data management at health facility level have been reported elsewhere [37, 38, 40]. However, the DHIS2 was limited in terms of ease of differentiating a zero report from missing data. These reports are difficult to interpret when retrieved from the system for lack of a clear display of “zero” to signify that no report was submitted at the given time [34].

Findings revealed predominant utilisation of paper-based reporting mechanisms at the lower surveillance levels. Similarly, most regional surveillance levels in Ghana received paper-based surveillance reports from the health facility level with very few instances of electronic reporting [34]. However, lack of standardised reporting channels resulted to inaccuracies in the reported data [34]. Therefore, use of electronic data transmission mechanisms would minimise the risk of surveillance inaccuracies across surveillance levels [34]. Surveillance systems face challenges relating to data inaccuracies as a consequence of data transmission challenges from the peripheral level [41]. Poor data quality minimises utilisation of the data for purposes of planning and decision-making [34]. Variations in data quality may be a factor of health workers’ reporting practices and attitudes towards surveillance activities [34]. Further findings indicated that there was inadequate training on surveillance data reporting. Consequently, lack of trained and computer-literate staff across the surveillance levels has ramifications on data quality [23, 39]. Regular supervision of surveillance reporting activities was lacking according to findings from our study. Notably, adequate support supervision ensures sustained capacity of health workers to collect quality surveillance data [39]. Inaccuracies in the weekly and monthly surveillance reports hampers utilisation of the data for planning and public health action [34, 42]. Therefore, policy makers are required to earmark funds to improve data quality within the surveillance system [34, 43]. Furthermore, countries are encouraged to heighten nationwide implementation of electronic surveillance systems to improve data quality [27]. Obtaining quality and reliable data requires deliberate partnership between donors, non-governmental organisations and health ministries in providing supervision and technical support at the primary health care level [44].

Most regional levels in Africa have previously either met or surpassed the recommended IDSR reporting timeliness threshold [45]. However, current study findings indicated that health facilities surveyed in PC-NTD endemic regions hardly met the 80% target reporting rate threshold. Only about a third of the health facilities met this threshold in terms of timeliness of reporting monthly surveillance data and slightly more than a half of the health facilities met the target threshold for completeness of monthly reports. Similarly, reporting timeliness and completeness rates were low in previous studies with findings indicating that health facilities failed to meet the 80% threshold for reporting rates [38, 41]. Nonetheless, elsewhere, the average reporting timeliness from the health facility to regional surveillance levels was higher than the target threshold [46]. Likewise, in a study conducted in an urban county in Kenya, reporting completeness and timeliness both exceeded the 80% IDSR reporting thresholds [47]. The current study scenario of low reporting rates could be attributed to data transmission challenges faced by health workers in the more remote PC-NTD endemic regions. Surveillance data timeliness is dependent on the surveillance level, objectives of the surveillance system and the activities and processes influencing data timeliness [48]. Similar to our study findings, reporting timeliness is hindered by the lack of appropriate and effective communication systems and equipment [38]. Therefore, data entry via DHIS2 and increased use of mobile phones to transmit surveillance data by lower level health facilities would improve reporting timeliness, data verification efforts and reduce logistical expenses resulting from hand-delivered reports [22]. Elsewhere, challenges facing timeliness of surveillance reports alluded to increased workload, poor telecommunication network coverage and lack of training [35]. Moreover, assessment of reporting rates is impeded by the lack of standardised methods for determining when reports are considered complete or timely [38].

Increased surveillance data timeliness and completeness were attributed to numerous channels of communication between the regional and higher surveillance levels. Moreover, enhanced technical support on IDSR implementation improved reporting rates [49]. Further comparable findings showed that improvements in reporting timeliness was ascribed to adoption of mobile phone messaging services that eased transmission of surveillance data [24]. Additionally, improved reporting timeliness resulted from increased sensitisation of health workers on the IDSR system [24]. Deliberate involvement by all health workers in surveillance data reporting would aid in high reporting rates concerning completeness and timeliness [38]. Moreover, providing designated surveillance staff at the regional levels of the health system would ensure adequate data reporting and management [38]. Reporting performance is often affected by circumstances where the person responsible for compilation and submission of the reports is unavailable. This identifies the need to involve all health workers in IDSR training and impart knowledge on the reporting requirements across all surveillance levels [38]. Furthermore, enhanced and continued training of surveillance staff would improve reporting accuracy, completeness and timeliness [35, 41, 45]. Notably, regular feedback across all surveillance levels positively influenced reporting timeliness and completeness [37, 50]. Further findings indicated that reporting rates for health facilities in PC-NTD endemic regions in Kenya declined in the 2017 surveillance period in comparison to the preceding two-year surveillance periods based on data retrieved from DHIS2 [28]. Comparably, the Global Health Observatory (GHO) data showed that Kenya had a surveillance index score of 50% in 2017, down from an index score of 85% in 2013 [51]. The decrease in index scores can be attributed to low performance of surveillance functions over the four-year period, however surveillance index scores for 2014, 2015 and 2016 for Kenya were unavailable from GHO data [51].

The mixed-study approach elicited respondents’ in depth perspectives on the surveillance system attributes. However, a limitation of the study was dependence on respondents’ self-reporting, which could have biased their responses due to social-desirability. Secondly, respondents’ perceptions of surveillance system attributes was limited to specific constructs as provided in the five-point likert scale questionnaire. However, a comment section was provided in the questionnaires enabling respondents to elucidate further on the attributes. Thirdly, fence sitting is a common shortcoming to using likert-type tools, hence the ‘not applicable’ and ‘don’t know’ options were provided to minimise this limitation [29]. Lastly, logistical challenges relating to inaccessible study sites, which were characterised by very poor terrain, harsh climate and community hostilities, were mitigated through use of mobile phone surveys as an alternative mechanism to obtain data from the respondents.

## Conclusions

The study depicted that surveillance system attributes in view of PC-NTDs hardly achieved the desired attribute scores (> 50) except for simplicity, acceptability and usefulness based on respondents’ perceptions. Health workers’ education level and years of work experience were socio-demographic characteristics mostly associated with respondents’ perceived opinion on the surveillance system attributes of acceptability and stability. On the other hand, health facility type and locality were facility characteristics associated with respondents’ perceived views on most surveillance system attributes. There was a notable decline in monthly reporting timeliness and completeness rates of the surveyed health facilities in the 2017 surveillance period. Ultimately, health workers’ perceptions on the surveillance system attributes influenced the overall performance of other surveillance core and support functions [25]. Therefore, the study findings should inform efforts centered on improving PC-NTDs surveillance and response in Kenya by tackling concerns of surveillance system users based on their perceptions towards surveillance system attributes.

## Supplementary Information

**Additional file 1: Supplementary file 1.** Health Personnel Questionnaire

**Additional file 2: Supplementary figure 1.** Simplicity of PC-NTDs surveillance system

**Additional file 3: Supplementary figure 2.** Acceptability of PC-NTDs surveillance system

**Additional file 4: Supplementary figure 3.** Stability of PC-NTDs surveillance system

**Additional file 5: Supplementary figure 4.** Flexibility of PC-NTDs surveillance system

**Additional file 6: Supplementary figure 5.** Usefulness of PC-NTDs surveillance system

**Additional file 7: Supplementary figure 6.** Data quality of PC-NTDs surveillance system

## Data Availability

The datasets generated and/or analysed to assess the surveillance system attributes are not publicly available due to the need to keep the identities of respondents confidential as they granted consent to be enrolled in the study on the basis of remaining anonymous, but are available from the corresponding author on reasonable request.

## Abbreviations

ARNTD: African Research Network for Neglected Tropical Diseases
COR-NTD: Coalition for Operational Research on Neglected Tropical Diseases
DHIS2: Demographic Health Information System II
GHO: Global Health Observatory
HFWs: Healthcare Facility Workers
IDSR: Integrated Diseases Surveillance and Response
IDSRU: Integrated Disease Surveillance and Response Unit
IQR: Inter-Quartile Range
KMO: Kaiser-Meyer Olkin
LF: Lymphatic Filariasis
NTDs: Neglected Tropical Diseases
PC-NTDs: Preventive Chemotherapy-targeted Neglected Tropical Diseases
STH: Soil Transmitted Helminthiasis
UK Aid: UK Aid from the British People
USA: United States of America
USAID: United States Agency for International Development
WHO: World Health Organization
