# Supplementary material for: Evaluation of health surveillance system attributes: the case of neglected tropical diseases in Kenya
Source: BMC Public Health. 2021 Feb 23;21:396. doi: 10.1186/s12889-021-10443-2 (PMC7903773; doi:10.1186/s12889-021-10443-2)
Supplement: Supplementary file 1 — Additional file 1: Supplementary file 1. Health Personnel Questionnaire [file 12889_2021_10443_MOESM1_ESM.docx]

**Supplementary File 1. Health Personnel Questionnaire**

| **GENERAL INFORMATION**  ***(Habari kwa ujumla)*** | | | | | | | | | |
| --- | --- | --- | --- | --- | --- | --- | --- | --- | --- |
| Region  *(Eneo)* | | | ………………………………………………. County  *(Kaunti)*  ……………………………………………….. Sub-County  *(Kaunti ndogo)* | | | | | | |
| Health facility name  (*Jina la kituo cha afya)* | | | ………………………………………………………………… | | | | | | |
| Demographic Characteristics  *(Demografia ya mhojiwa)* | | | Age *(Umri)* | | □ 18-30 □ 31-40  □ 41-50 □ >50 | | | | |
|  |  |  | Sex *(Jinsia)* | | Male *(Kiume)* □ Female *(Kike)* □ | | | | |
| What is your health cadre?  *(Wajibu wa mfanyakazi wa afya)* | | | □ Medical Practitioner *(Matibabu daktari)*  □ Clinical Officer *(Afisa wa kliniki)*  □ Nurse *(Muuguzi)*  □ Public Health Staff *(Mfanyakazi wa afya ya umma)*  □ Laboratory Staff *(Mfanyakazi wa maabara)*  □ Health Records Management Staff *(Mfanyakazi wa kumbukumbu za afya)*  □ Other Cadre *(Uajibu zinginezo):*…………………………… | | | | | | |
| Number of years worked in health cadre  *(Miaka yako kikazi katika uajibu huu?)* | | | □ Less than a year *(Chini ya mwaka 1)*  □ 1-2 years *(Kati ya mwaka 1-2)*  □ 2-3 years *(Kati ya miaka 2-3)*  □ 3-5 years *(Kati ya miaka 3-5)*  □ More than 5years *(Zaidi ya miaka 5)* | | | | | | |
| What is your highest level of education?  *(Kiwango chako cha juu zaidi katika masomo?)* | | | □ PhD *(Shahada ya uzamifu)*  □ Masters *(Shahada ya uzamili)*  □ Degree *(Shahada)*  □ Diploma *(Stashahada)*  □ Certificate *(Astashahada)* | | | | | | |
| **(Tick (√) in the most appropriate box & please complete the “comments” section for each attribute)**  ***(Weka alama ya tiki (√) pahali panapofaa zaidi na pia toa maoni yako kwa kila sifa wa mfumo)*** | | | | | | | | | |
| **SIMPLICITY *(URAHISI WA MFUMO)*** | | | | | | | | | |
|  | ***Strongly Disagree***  ***(Sikubali zaidi)*** | ***Disagree***  ***(Sikubali)*** | | ***Neither Agree or Disagree***  ***(Sikubali wala Sikatai)*** | | ***Agree***  ***(Na kubali)*** | ***Strongly***  ***Agree***  ***(Na kubali zaidi)*** | ***Not***  ***Applic-able***  ***(Si husika)*** | ***Don’t***  ***Know***  ***(Sijui)*** |
| **Q1.** Reporting of PC-NTDs surveillance data within the IDSR system is easy.  *(Kuripoti kwa magonjwa ya kitropiki yaliyo telekezwa kupitia mfumo wa IDSR ni rahisi)* |  |  | |  | |  |  |  |  |
| **Q2.** Forms for reporting PC-NTDs surveillance data are easy to complete.  *(Fomu za kuripoti magonjwa ya kitropiki yaliyo telekezwa ni rahisi kukamilisha)* |  |  | |  | |  |  |  |  |
| **Q3.** Instructions and guidelines for completing the PC-NTDs reporting forms are easy to understand.  *(Maelekezo na miongozo za kukamilisha fomu za kuripoti* *magonjwa ya kitropiki yaliyo telekezwa ni rahisi kuelewa)* |  |  | |  | |  |  |  |  |
| **Q4.** Understanding the functionality of the IDSR system is easy.  *(Kuelewa utendaji wa mfumo wa IDSR ni rahisi)* |  |  | |  | |  |  |  |  |
| **Q5.** All health personnel are conversant with the IDSR system.  *(Wafanyakazi wote wa afya wanaelewa mfumo huu wa IDSR)* |  |  | |  | |  |  |  |  |
| **Q6.** Case definitions for PC-NTDs are easily applicable.  *(Miongozo za kutambua kesi za magonjwa ya kitropiki yaliyo telekezwa hutumika kwa urahisi)* |  |  | |  | |  |  |  |  |
| **Q7.** PC-NTD surveillance data is easily managed.  *(Data za ufuatiliaji wa magonjwa ya kitropiki yaliyo telekezwa ni rahisi kuandaa)* |  |  | |  | |  |  |  |  |
| **Q8.** The existing IDSR system easily accommodates all PC-NTDs.  *(Mfumo uliopo wa IDSR unaweza kujumuisha magonjwa yote ya kitropiki yaliyo telekezwa kwa urahisi)* |  |  | |  | |  |  |  |  |
| **Q9.** Methods used for collection of PC-NTDs surveillance data are simple.  *(Mbinu za ukasanyaji wa data za ufuatiliaji wa magonjwa ya kitropiki yaliyo telekezwa ni rahisi)* |  |  | |  | |  |  |  |  |
| **Q10.** Time spent collecting PC-NTDs surveillance data is minimal.  *(Muda unaotumika kukusanya data za ufuatiliaji wa magonjwa ya kitropiki yaliyo telekezwa ni fupi)* |  |  | |  | |  |  |  |  |
| **Q11.** Methods used for analysis of PC-NTDs surveillance data are simple.  *(Mbinu zinazotumika kuchanganua habari za magonjwa ya kitropiki yaliyo telekezwa ni rahisi)* |  |  | |  | |  |  |  |  |
| **Q12.** Time spent in analysis of PC-NTDs surveillance data is minimal.  *(Muda unaotumika kuchanganua habari za magonjwa ya kitropiki yaliyo telekezwa ni fupi)* |  |  | |  | |  |  |  |  |
| **Q13.** Minimal training is required to manage PC-NTDs surveillance data.  *(Mafunzo machache yanahitajika kutimiza shughuli za usimamizi* *wa* *magonjwa ya kitropiki yaliyo telekezwa)* |  |  | |  | |  |  |  |  |
| **Q14.** The follow-up process for PC-NTDs surveillance data is simple.  *(Mchakato wa ufuatiliaji wa* *habari ya* *magonjwa ya kitropiki yaliyo telekezwa ni rahisi)* |  |  | |  | |  |  |  |  |
| **Q15.** The reporting levels for PC-NTDs surveillance data are minimal.  *(Viwango vya kuripoti habari za* *magonjwa ya kitropiki yaliyo telekezwa ni chache)* |  |  | |  | |  |  |  |  |
| **Comments *(Maoni)*:** | | | | | | | | | |
| **ACCEPTABILITY *(UKUBALIKAJI WA MFUMO)*** | | | | | | | | | |
|  | ***Strongly Disagree***  ***(Sikubali zaidi)*** | ***Disagree***  ***(Sikubali)*** | | ***Neither Agree or Disagree***  ***(Sikubali wala Sikatai)*** | | ***Agree***  ***(Na kubali)*** | ***Strongly***  ***Agree***  ***(Na kubali zaidi)*** | ***Not***  ***Applic-able***  ***(Si husika)*** | ***Don’t***  ***Know***  ***(Sijui)*** |
| **Q1.** My contribution/s and input/s to the existing IDSR system is/are considered valuable.  *(Mchango wangu unathaminika katika mfumo wa IDSR uliopo kwa mkoa huu)* |  |  | |  | |  |  |  |  |
| **Q2.** I am satisfied with my involvement in PC-NTDs surveillance activities in this facility.  *(Nimeridhishwa na kuhusika kwangu kwa shughuli za ufuatiliaji wa* *magonjwa ya kitropiki yaliyo telekezwa)* |  |  | |  | |  |  |  |  |
| **Q3.** Fellow health personnel in this facility show interest in PC-NTDs surveillance activities.  *(Wafanyakazi wenza katika kituo hiki cha afya wanahamu za kuhusishwa na shughuli za ufuatiliaji* wa *magonjwa ya kitropiki yaliyo telekezwa)* |  |  | |  | |  |  |  |  |
| **Q4.** All actions regarding PC-NTDs surveillance are adequately supported by the health facility management.  *(Shughuli za ufuatiliaji wa* *magonjwa ya kitropiki yaliyo telekezwa yanaungwa mkono na usimamizi wa kituo hiki cha afya)* |  |  | |  | |  |  |  |  |
| **Q5.** PC-NTDs are considered of public health importance in the region.  *(Magonjwa ya kitropiki yaliyo telekezwa yanapewa umuhimu katika afya ya umma wa mkoa huu)* |  |  | |  | |  |  |  |  |
| **Q6.** The community in this region supports PC-NTDs surveillance activities undertaken by this facility.  *(Jamii kwa ujumla katika mkoa huu inaunga mkono shughuli za ufuatiliaji wa magonjwa ya kitropiki yaliyo telekezwa katika kituo hiki cha afya)* |  |  | |  | |  |  |  |  |
| **Q7.** The existing IDSR system protects users’ privacy and confidentiality.  *(Mfumo wa IDSR inalinda faragha na siri za watumizi wote wa mfumo huu)* |  |  | |  | |  |  |  |  |
| **Comments *(Maoni)*:** | | | | | | | | | |
| **STABILITY *(UIMARA WA MFUMO)*** | | | | | | | | | |
|  | **Strongly Disagree**  ***(Sikubali zaidi)*** | **Disagree**  ***(Sikubali)*** | | **Neither Agree or Disagree**  ***(Sikubali wala Sikatai)*** | | **Agree**  ***(Na kubali)*** | **Strongly**  **Agree**  ***(Na kubali zaidi)*** | ***Not***  ***Applic-able***  ***(Si husika)*** | **Don’t**  **Know**  ***(Sijui)*** |
| **Q1.** The existing IDSR system has always been reliable when reporting PC-NTDs surveillance data.  *(Mfumo wa IDSR ni wa kuaminika wakati wote kwa kuripoti magonjwa ya kitropiki yaliyo telekezwa)* |  |  | |  | |  |  |  |  |
| **Q2.** PC-NTDs surveillance reporting forms are always available when required.  *(Fomu za kuripoti magonjwa ya kitropiki yaliyo telekezwa hupatikana kwa urahisi zinapo hitajika)* |  |  | |  | |  |  |  |  |
| **Q3.** Problems experienced within the IDSR system regarding PC-NTDs surveillance are addressed with minimal delays.  *(Changamoto zozote zinazo husu ufuatiliaji wa magonjwa ya kitropiki yaliyo telekezwa kupitia kwa mfumo wa IDSR zinashughulikiwa kwa haraka)* |  |  | |  | |  |  |  |  |
| **Q4.** PC-NTDs surveillance and response is well supported by those overseeing disease surveillance and response activities in this region/facility.  *(Shughuli za ufuatiliaji wa magonjwa ya kitropiki yaliyo telekezwa zinaungwa mkono na wasimamizi wa shughuli hizi katika mkoa huu na kituo hiki cha afya)* |  |  | |  | |  |  |  |  |
| **Q5.** Resources provided for PC-NTDs surveillance and response activities in this region/facility are sufficient.  *(Rasilimali zinazotolewa katika shughuli za ufuatiliaji wa magonjwa ya kitropiki yaliyo telekezwa kwa mkoa huu ni za kutosha)* |  |  | |  | |  |  |  |  |
| **Q6.** PC-NTDs surveillance data and records storage in this facility is safe and efficient.  *(Kumbukumbu za habari za ufuatiliaji wa magonjwa ya kitropiki yaliyo telekezwa zinalindwa vyema)* |  |  | |  | |  |  |  |  |
| **Comments *(Maoni)*:** | | | | | | | | | |
| **FLEXIBILITY *(UBADILISHIKAJI WA MFUMO)*** | | | | | | | | | |
|  | **Strongly Disagree**  ***(Sikubali zaidi)*** | **Disagree**  ***(Sikubali)*** | | **Neither Agree or Disagree**  ***(Sikubali wala Sikatai)*** | | **Agree**  ***(Na kubali)*** | **Strongly**  **Agree**  ***(Na kubali zaidi)*** | ***Not***  ***Applic-able***  ***(Si husika)*** | **Don’t**  **Know**  ***(Sijui)*** |
| **Q1.** The existing surveillance reporting system is well adapted to reporting all the endemic PC-NTDs in this region.  *(Mfumo wa ufuatiliaji wa magonjwa uliopo katika mkoa huu inachukulia vyema kuripoti kwa* *magonjwa ya kitropiki yaliyo telekezwa)* |  |  | |  | |  |  |  |  |
| **Q2.**PC-NTDs surveillance and response is efficiently achieved within the IDSR system.  *(Ufuatiliaji wa magonjwa ya kitropiki yaliyo telekezwa inafanikishwa kwa ufanisi kupitia mfumo wa IDSR)* |  |  | |  | |  |  |  |  |
| **Q3.** The existing IDSR system easily adapts to changes in PC-NTDs information needs (e.g. case definitions)  *(Mfumo wa IDSR inachukulia vyema mabadiliko ya mahitaji ya habari za magonjwa ya kitropiki yaliyo telekezwa kama vile kubaini kesi za magonjwa hizi)* |  |  | |  | |  |  |  |  |
| **Q4.** PC-NTDs surveillance and response within the existing IDSR system easily adapts to changes in funding in the region  *(Ufuatiliaji wa magonjwa ya kitropiki yaliyo telekezwa kupitia mfumo wa IDSR inachukulia kwa urahisi mabadiliko za fedha katika mkoa huu)* |  |  | |  | |  |  |  |  |
| **Q5.** PC-NTDs surveillance and response within the existing IDSR system easily adapts to changes in technology (e.g. paper-based to electronic-based reporting)  *(Ufuatiliaji wa magonjwa ya kitropiki yaliyo telekezwa kupitia mfumo wa IDSR inachukulia kwa urahisi mabadiliko ya kiteknologia)* |  |  | |  | |  |  |  |  |
| **Q6.** The existing IDSR system easily adapts to new PC-NTDs surveillance data sources  *(Mfumo wa IDSR uliopo inachukulia kwa urahisi mianzo mapya ya habari za magonjwa ya kitropiki yaliyo telekezwa)* |  |  | |  | |  |  |  |  |
| **Comments *(Maoni)*:** | | | | | | | | | |
| **USEFULNESS *(UMANUFAA WA MFUMO)*** | | | | | | | | | |
|  | **Strongly Disagree**  ***(Sikubali zaidi)*** | **Disagree**  ***(Sikubali)*** | | **Neither Agree or Disagree**  ***(Sikubali wala Sikatai)*** | | **Agree**  ***(Na kubali)*** | **Strongly**  **Agree**  ***(Na kubali zaidi)*** | ***Not***  ***Applic-able***  ***(Si husika)*** | **Don’t**  **Know**  ***(Sijui)*** |
| **Q1.** PC-NTDs surveillance and response within the IDSR system has enabled achievement of the surveillance objectives in the past one year in this region.  *(Ufuatiliaji wa* *magonjwa ya kitropiki yaliyo telekezwa iliwezesha mafanikio ya malengo yaliyowekwa kwa mwaka uliopita )* |  |  | |  | |  |  |  |  |
| **Q2.** PC-NTDs surveillance data has informed program implementation for control of the diseases in the past one year in this region.  *(Ufuatiliaji wa* *magonjwa ya kitropiki yaliyo telekezwa umewezesha utekelezaji wa uratibu kwa madhumuni ya kudhibiti magonjwa haya)* |  |  | |  | |  |  |  |  |
| **Q3.** PC-NTDs surveillance data generated within the IDSR system has stimulated research activities in this region.  *(Ufuatiliaji wa* *magonjwa ya kitropiki yaliyo telekezwa kupitia kwa mfumo wa IDSR imewezesha shughuli za utafiti katika mkoa huu)* |  |  | |  | |  |  |  |  |
| **Q4.** PC-NTDs surveillance data generated within the IDSR system has attracted donor funding for disease control in this region.  *(Ufuatiliaji wa* *magonjwa ya kitropiki yaliyo telekezwa kupitia mfumo wa IDSR imevutia fedha kutoka kwa wafadhili katika mkoa huu)* |  |  | |  | |  |  |  |  |
| **Q5.** PC-NTDs surveillance and response activities in this region are considered important within the IDSR system.  *(Shughuli za* *ufuatiliaji wa* *magonjwa ya kitropiki yaliyo telekezwa katika mkoa huu zinachukulika kuwa za muhimu kupitia mfumo wa IDSR)* |  |  | |  | |  |  |  |  |
| **Q6.** The IDSR system provides sufficient information for prompt public health action to PC-NTDs in this region.  *(Mfumo wa IDSR inatoa habari za kutosha kuwezesha hatua za afya ya umma kuchukuliwa kulenga magonjwa ya kitropiki yaliyo telekezwa katika mkoa huu)* |  |  | |  | |  |  |  |  |
| **Q7.** PC-NTDs surveillance data generated within the IDSR system provides an estimate of morbidity magnitude in this region.  *(Habari za ufuatiliaji wa* *magonjwa ya kitropiki yaliyo telekezwa* *kupitia mfumo wa IDSR zinaeleza ukubwa ya makisio ya magonjwa haya katika mkoa huu)* |  |  | |  | |  |  |  |  |
| **Q8.** PC-NTDs surveillance data identifies risk factors associated with the cases reported.  *(Habari za ufuatiliaji wa* *magonjwa ya kitropiki yaliyo telekezwa yanawezesha utambuzi wa hatari ya magonjwa haya)* |  |  | |  | |  |  |  |  |
| **Q9.** PC-NTDs surveillance data generated within the IDSR system detects trends in changes of case occurrence in this region.  *(Habari za ufuatiliaji wa* *magonjwa ya kitropiki yaliyo telekezwa kupitia mfumo wa IDSR inawezesha utambuzi wa mienendo ya mabadiliko ya tukio ya magonjwa haya katika mkoa huu)* |  |  | |  | |  |  |  |  |
| **Q10.** PC-NTDs **s**urveillance data enables prevention and control programmes impact assessment in this region.  *(Habari za ufuatiliaji wa* *magonjwa ya kitropiki yaliyo telekezwa zinawezesha tathmini za athari ya miradi ya kuzuia na kudhibiti magonjwa haya)* |  |  | |  | |  |  |  |  |
| **Q11.** PC-NTDs surveillance data generated within the IDSR system has an impact on the clinical diagnosis practices.  *(Habari za ufuatiliaji wa* *magonjwa ya kitropiki yaliyo telekezwa kupitia mfumo wa IDSR zinaathiri shughuli za utambuzi wa kikliniki kuhusu magonjwa hayo)* |  |  | |  | |  |  |  |  |
| **Comments *(Maoni)*:** | | | | | | | | | |
| **DATA QUALITY *(UBORA WA HABARI)*** | | | | | | | | | |
|  | **Strongly Disagree**  ***(Sikubali zaidi)*** | **Disagree**  ***(Sikubali)*** | | **Neither Agree or Disagree**  ***(Sikubali wala Sikatai)*** | | **Agree**  ***(Na kubali)*** | **Strongly**  **Agree**  ***(Na kubali zaidi)*** | ***Not***  ***Applic-able***  ***(Si husika)*** | **Don’t**  **Know**  ***(Sijui)*** |
| **Q1.** Missing data is a common occurrence in PC-NTDs surveillance reporting forms in this region.  *(Ukosefu wa habari ni tukio la kawaida kwa fomu zinazotumika kuripoti magonjwa ya kitropiki yaliyo telekezwa katika mkoa huu)* |  |  | |  | |  |  |  |  |
| **Q2.** Surveillance electronic/hardcopy forms for reporting PC-NTDs are clear and elaborate.  (*Fomu za kuripoti magonjwa ya kitropiki yaliyo telekezwa ni wazi na zenye ufafanuzi tosha)* |  |  | |  | |  |  |  |  |
| **Q3.** Training offered regarding completion of PC-NTDs surveillance reporting forms is adequate.  *(Mafunzo yanayo tolewa kuhusu ukamilishaji wa* *fomu hizi za kuripoti magonjwa ya kitropiki yaliyo telekezwa ni ya kutosha)* |  |  | |  | |  |  |  |  |
| **Q4.** Supervision offered during PC-NTDs surveillance reporting forms completion is adequate  *(Usimamizi unaotolewa wakati wa ukamilishaji wa* *fomu hizi za kuripoti magonjwa ya kitropiki yaliyo telekezwa ni wa kutosha)* |  |  | |  | |  |  |  |  |
| **Q5.** Time allocated for PC-NTDs surveillance data management is adequate  *(Muda unaotolewa kukamilisha shughuli za uchanganuzi na usimamizi wa habari za* *magonjwa ya kitropiki yaliyo telekezwa ni wa kutosha)* |  |  | |  | |  |  |  |  |
| **Comments *(Maoni)*:** | | | | | | | | | |
