# Supplementary figures and images for: Evaluation of health surveillance system attributes: the case of neglected tropical diseases in Kenya
Source: BMC Public Health. 2021 Feb 23;21:396. doi: 10.1186/s12889-021-10443-2 (PMC7903773; doi:10.1186/s12889-021-10443-2)

**Supplementary figure 1. Simplicity of PC-NTDs surveillance system**


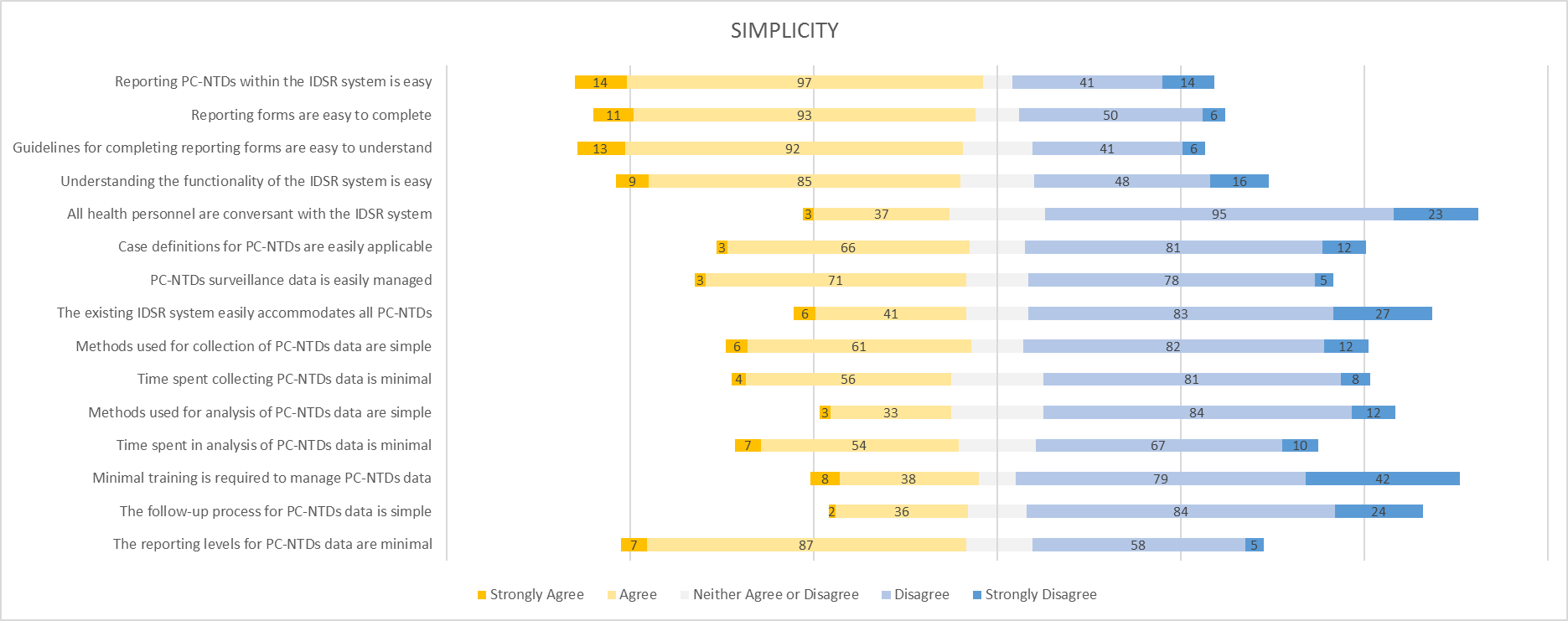

Supplement: Supplementary file 2 — Additional file 2: Supplementary figure 1. Simplicity of PC-NTDs surveillance system [file 12889_2021_10443_MOESM2_ESM.docx]

**Supplementary figure 2. Acceptability of PC-NTDs surveillance system**


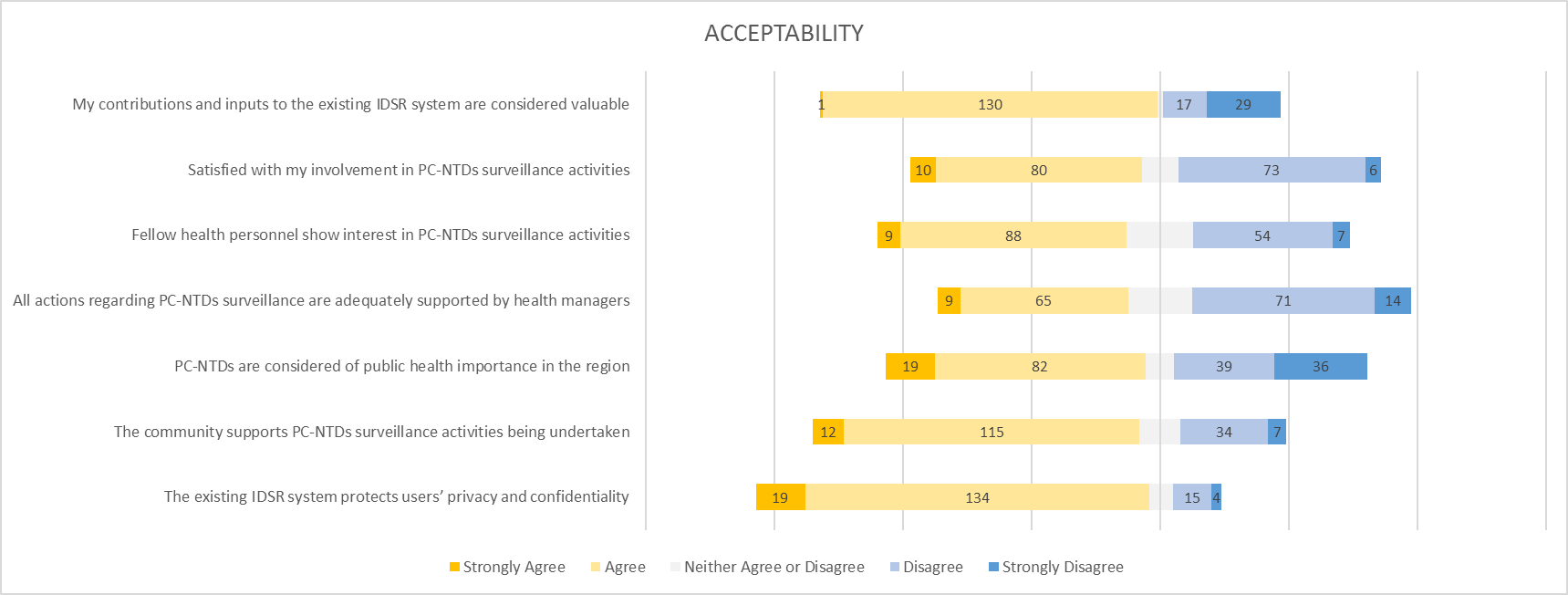

Supplement: Supplementary file 3 — Additional file 3: Supplementary figure 2. Acceptability of PC-NTDs surveillance system [file 12889_2021_10443_MOESM3_ESM.docx]

**Supplementary figure 3. Stability of PC-NTDs surveillance system**


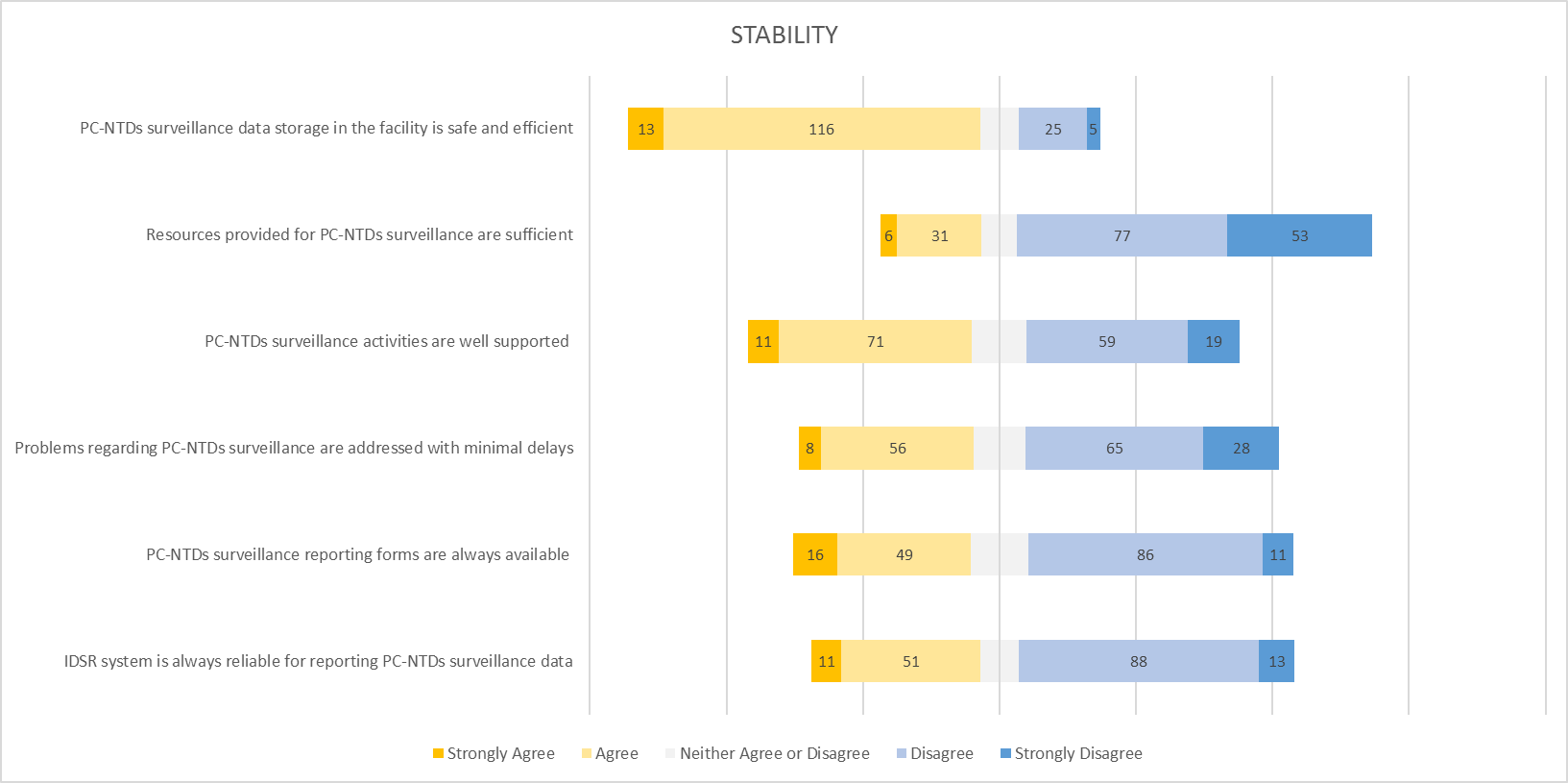

Supplement: Supplementary file 4 — Additional file 4: Supplementary figure 3. Stability of PC-NTDs surveillance system [file 12889_2021_10443_MOESM4_ESM.docx]

**Supplementary figure 4. Flexibility of PC-NTDs surveillance system**


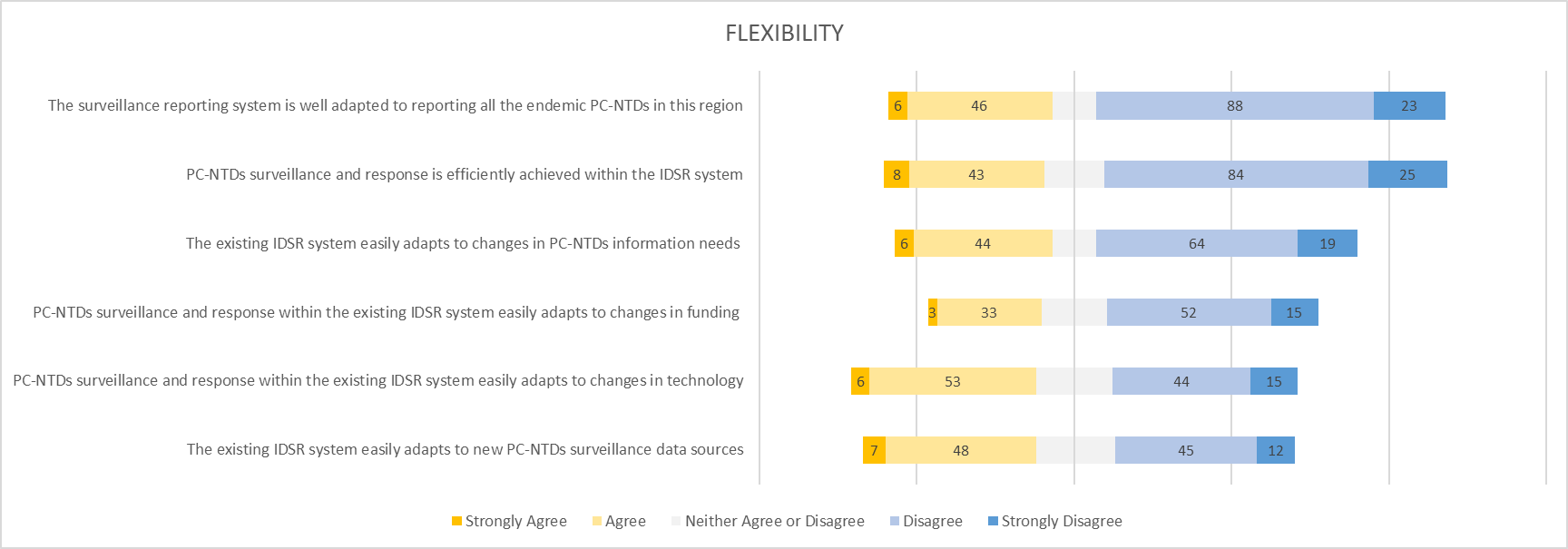

Supplement: Supplementary file 5 — Additional file 5: Supplementary figure 4. Flexibility of PC-NTDs surveillance system [file 12889_2021_10443_MOESM5_ESM.docx]

**Supplementary figure 5. Usefulness of PC-NTDs surveillance system**


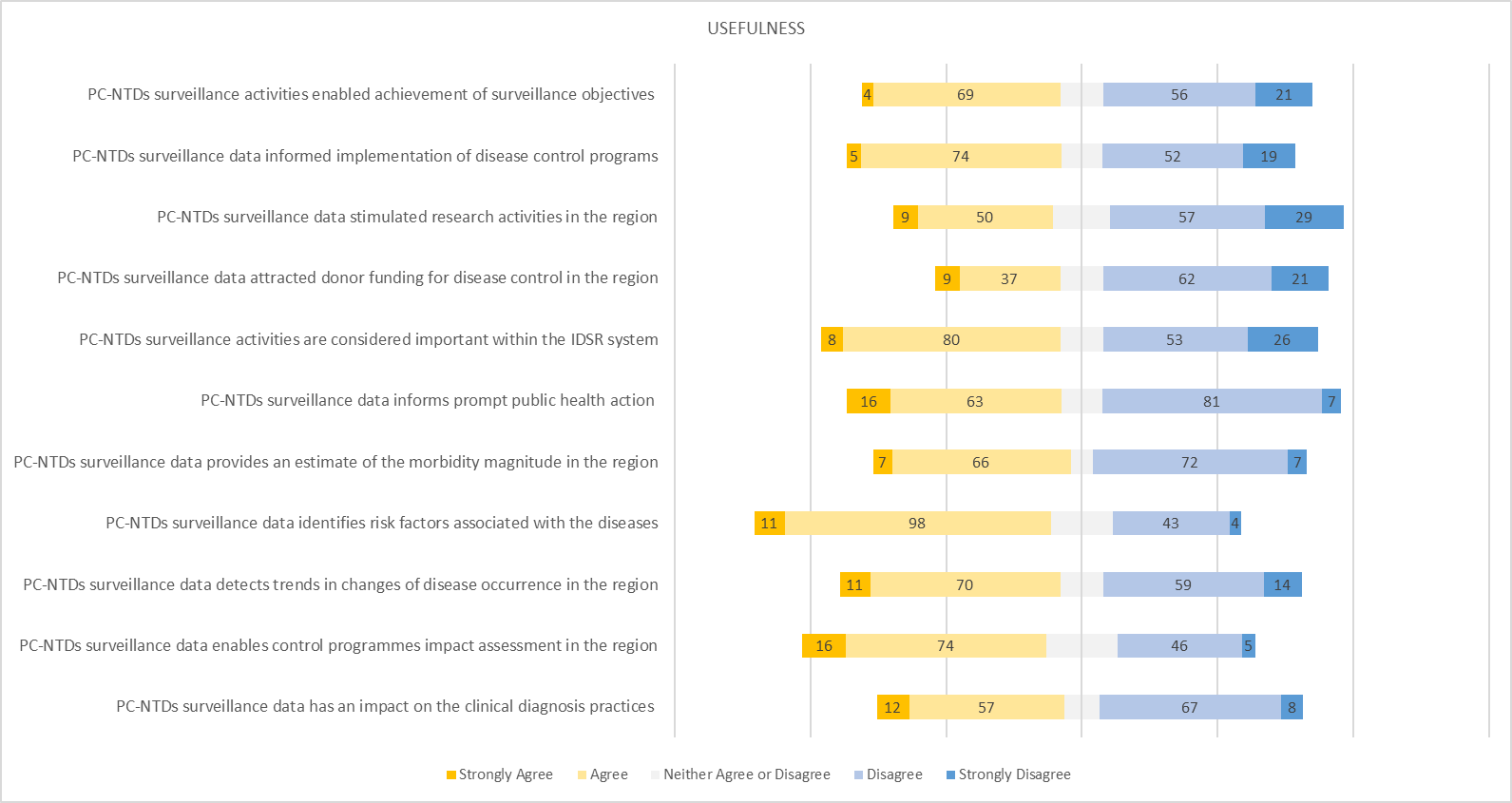

Supplement: Supplementary file 6 — Additional file 6: Supplementary figure 5. Usefulness of PC-NTDs surveillance system [file 12889_2021_10443_MOESM6_ESM.docx]

**Supplementary figure 6. Data quality of PC-NTDs surveillance system**


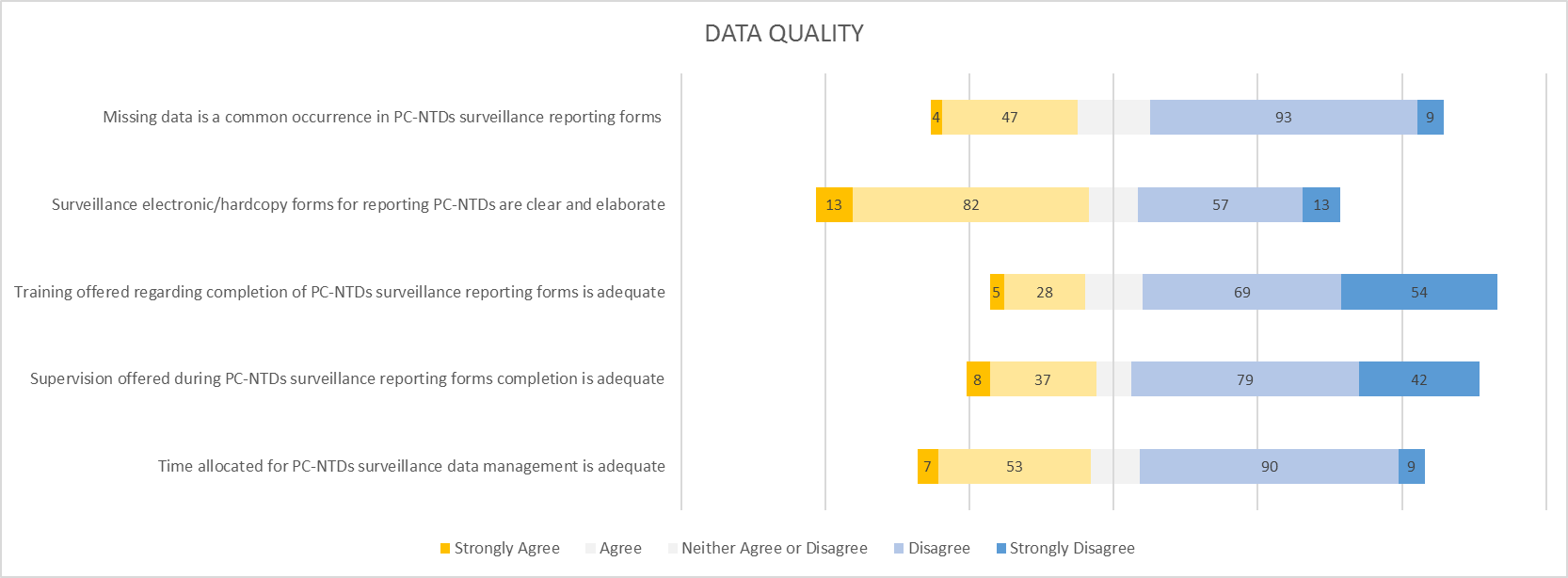

Supplement: Supplementary file 7 — Additional file 7: Supplementary figure 6. Data quality of PC-NTDs surveillance system [file 12889_2021_10443_MOESM7_ESM.docx]
